# Incoordination between spikes and LFPs in Aβ_1−42_-mediated memory deficits in rats

**DOI:** 10.3389/fnbeh.2014.00411

**Published:** 2014-11-27

**Authors:** Wenwen Bai, Hu Yi, Tiaotiao Liu, Jing Wei, Xin Tian

**Affiliations:** Department of Biomedical Engineering, School of Biomedical Engineering and Technology, Tianjin Medical UniversityTianjin, China

**Keywords:** Alzheimer's disease (AD), working memory, spike-LFP coordination, entropy correlation, rat

## Abstract

Alzheimer's disease (AD) is a neurodegenerative disease that gradually induces cognitive deficits. Impairments of working memory have been typically observed in AD. It is well known that spikes and local field potentials (LFPs) as well as the coordination between them encode information in normal brain function. However, the abnormal coordination between spikes and LFPs in the cognitive deficits of AD has remained largely unexplored. As amyloid-β peptide (Aβ) is a causative factor for the cognitive impairments of AD, developing a mechanistic understanding of the contribution of Aβ to cognitive impairments may yield important insights into the pathophysiology of AD. In the present study, we simultaneously recorded spikes and LFPs from multiple electrodes implanted in the prefrontal cortex of rats (control and intra-hippocampal Aβ injection group) that performed a Y-maze working memory task. The information changes in spikes and LFPs during the task were assessed by calculation of entropy. Then the coordination between spikes and LFPs was estimated by the correlation of LFP entropy and spike entropy. Compared with the control group, the Aβ group showed significantly weaker coordination between spikes and LFPs. Our results indicate that the incoordination between spikes and LFPs may provide a potential mechanism for the cognitive deficits in working memory of AD.

## Introduction

Alzheimer's disease (AD), the most common cause of dementia in the elderly, is a neurodegenerative disorder that gradually induces cognitive deficit (Haffen et al., 2011). Impairment of working memory, in particular, is typically observed in AD (Baddeley et al., 1991; Kensinger et al., 2003). Working memory—a system for the temporary holding and manipulation of information—is important for a range of cognitive tasks such as learning, comprehension and reasoning (Baddeley, 1986, 2010). The research on working memory deficit is beneficial for lifting the veil of the mechanisms underlying memory deficits in AD.

Accumulating evidence has identified prefrontal cortex (PFC) as playing a critical role in working memory (Baddeley et al., 2000; Fuster, 2001; Dalley et al., 2004; Vertes, 2006; Horst and Laubach, 2009). Hippocampus is also an essential structure for working memory. Inactivation or lesion to the hippocampus produces a severe deficit in working memory (Steele and Morris, 1999; Bannerman et al., 2008; O'Neill et al., 2013; Zhang et al., 2013). Furthermore, neural activity in the PFC becomes synchronized with activity in the hippocampus during working memory tasks, and the strength of hippocampal-PFC synchrony is correlated with animals' behavioral performance (Jones and Wilson, 2005; Hyman et al., 2010; Sigurdsson et al., 2010). Lesion studies have suggested such hippocampal-PFC interaction is critical for successful task performance (Izaki et al., 2008). Since Aβ is a causative factor for the cognitive impairments in AD, we expect to develop a mechanistic understanding of the effects of intra-hippocampal Aβ on PFC, which may yield important insights into the pathophysiology of AD.

Much of our mechanistic understanding of brain function comes from extracellular recordings. The neural signals recorded with extracellular microelectrodes—commonly decomposed into spikes and local field potentials (LFPs)—are measurements for studying the spatiotemporal organization of information processing circuits underlying learning and memory. Accumulated evidence has suggested that spikes and LFPs in cortical circuits encode information in cognitive processes (Pesaran et al., 2002; Baeg et al., 2003; Lawhern et al., 2011; Li et al., 2012) as well as cooperatively encode cognitive information (Lee et al., 2005; Berens et al., 2008; Zanos et al., 2012). Elucidating the relationship between spikes and LFPs therefore has important implications for understanding the properties of *in vivo* cortical neurons, the links between single-cell and network activity, and the organization of cortical circuits (Zanos et al., 2012).

Popular methods for studying spike-LFP coordination include spike-triggered averaging, spike-field coherence and phase synchronization. These analyses have been proven useful for inferring the role of feedforward and feedback circuitry in functions as diverse as memory, perception, attention, and motor control (Jacobs et al., 2007; Chalk et al., 2010; Saleh et al., 2010; Ray and Maunsell, 2011; Zanos et al., 2011). Since neural responses are generally non-linear, information entropy was proposed as useful tool for extracting the non-linear characteristics of neural responses in cognitive functions. The entropy of a random variable is defined in terms of its probability distribution and can be shown to be a good measure of the randomness or the uncertainty (Strong et al., 1998; Quiroga and Panzeri, 2009). Entropy has been widely applied to analyze electrophysiological data and explore neural information characteristics (Rosso, 2007; Belitski et al., 2008; Kayser et al., 2009; Dorval, 2011). In particular, entropy has been used for the analysis of EEGs and MEGs in AD (Dauwels et al., 2010; Gómez and Hornero, 2010; Mizuno et al., 2010; Bruña et al., 2012). A growing body of research indicates that entropy-based analytic methods may be effective approaches for characterizing and understanding abnormal cortical dynamics in AD (Tsai et al., 2012; Chen and Pham, 2013; Yang et al., 2013; McBride et al., 2014). Our previous study has revealed strengthened spike-LFP coordination during working memory tasks in healthy subjects with normal brain function (Li et al., 2014). It is reasonable to study the spike-LFP coordination in working memory deficit, because the abnormal coordination between spikes and LFPs may be a candidate mechanism for the pathological progression of AD.

Therefore, in the present study, we simultaneously recorded the spikes and LFPs in the PFC of rats (normal and intra-hippocampal Aβ injection groups) while the rats performed a Y-maze working memory task. We then examined the coordination between the spikes and LFPs based on Shannon entropy, to study how the spikes and LFPs cooperatively encode information during the working memory task and obtain insights into how abnormal neural coordination could contribute to the memory deficits in AD.

## Materials and methods

All surgical and experimental procedures conformed to the Guide for the Care and Use of Laboratory Animals and approved by the Tianjin Medical University Animal Care and Use Committee. The drug usage in the experiments complied with the Chinese Pharmacopeia (2010 edition), approved by Chinese Pharmacopeia Commission.

### Subjects

Sprague-Dawley rats (male, 300–350 g, 12–14 weeks) were obtained from the Experimental Animal Center of Tianjin Medical University. Rats were housed in plastic cages (3–4 per cage) in a climate-controlled room (24°C) with a 12 h light/12 h dark cycle. Food and water were available *ad libitum*. The rats were divided randomly into two groups: Group-I (Aβ group) was comprised of Aβ_1−42_-induced toxicity rats, bilaterally injected with Aβ_1−42_ in the dentate gyrus (DG) area of dorsal hippocampus; Group-II (control group) was comprised of healthy rats, bilaterally injected with saline (pH 7.4).

### Aβ_1−42_-induced toxicity rat model

Diverse lines of evidence suggested that Aβ deposition has a causal role in inducing neuronal dysfunction and cognitive decline in AD (Palop and Mucke, 2010; Karran et al., 2011). In previous studies, intra-hippocampal injection with Aβ in rodents has been widely used as a model for AD since measurable Aβ deposition is associated with persistent memory decline (Nomura et al., 2012). The details of model establishment have been described previously (Tan et al., 2013).

Aβ_1−42_ peptide (Sigma, USA) was prepared from 1 μg/μl soluble Aβ_1−42_ solution, which was dissolved in filtered phosphate buffered saline (PBS: 10 mM NaH2PO4 \ Na2HPO4, 100mM NaCl, dissolved in glass-distilled deionized water, pH = 7.5). Aβ_1−42_ solution was then incubated under vigorous agitation using a Tefion-coated stirbar at 23°C for 36 h. Then, the incubated Aβ_1−42_ solution was ready for injection. For Aβ_1−42_ injection, the rats were anesthetized with chloral hydrate (300 mg/kg, i.p.) and placed in a stereotaxic apparatus. Incubated Aβ_1−42_ (5 μl, 1 μg/μl) was injected into DG of dorsal hippocampus (dHPC) bilaterally (anterior posterior, 3.2 mm; lateral, 2.5 mm; horizontal, 3.5 mm from bregma) (Christensen et al., 2008). Rats with subsequent memory deficits as identified using a Y-maze test were considered to have Aβ_1−42_-induced toxicity model.

### Immunohistochemistry

We investigated whether the incubated Aβ_1−42_ had been successfully injected into the hippocampus in the Aβ_1−42_injection group using immunohistochemistry, as described previously (Tan et al., 2013). Rats were deeply anesthetized and transcardially perfused with PBS (0.01 mol/l) and fixative (4% paraformaldehyde, 0.2% picric acid, diluted in 0.1 mol/l phosphate buffer, pH = 7.4). Rat brains were dissected and post-fixed in 4% PFA (diluted in 0.1 mol/l PB buffer) for 24 h at 4°C. Brains were embedded in paraffin and cut into 5-μm coronal sections. The sections were then dewaxed in xylene and rehydrated in a series of graded alcohols according to histopathological standards. To remove residual peroxidase activity, the sections were treated with H2O2 (3%, for 30 min) and rinsed with PBS. Microwave antigen retrieval was applied with slides immersed in 10 mM citrate buffer (pH = 6.0). Slides were blocked with 10% normal goat serum, and incubated with rabbit polyclonal anti-Aβ_1−42_ antibody (1:250, Abcam 10148) at 4°C overnight and then incubated with corresponding biotinylated secondary antibodies (1:200). The immunoreactivity was developed using DAB for 3–10 mins.

### Delayed-alternation task in Y-maze

**Figure 1:**
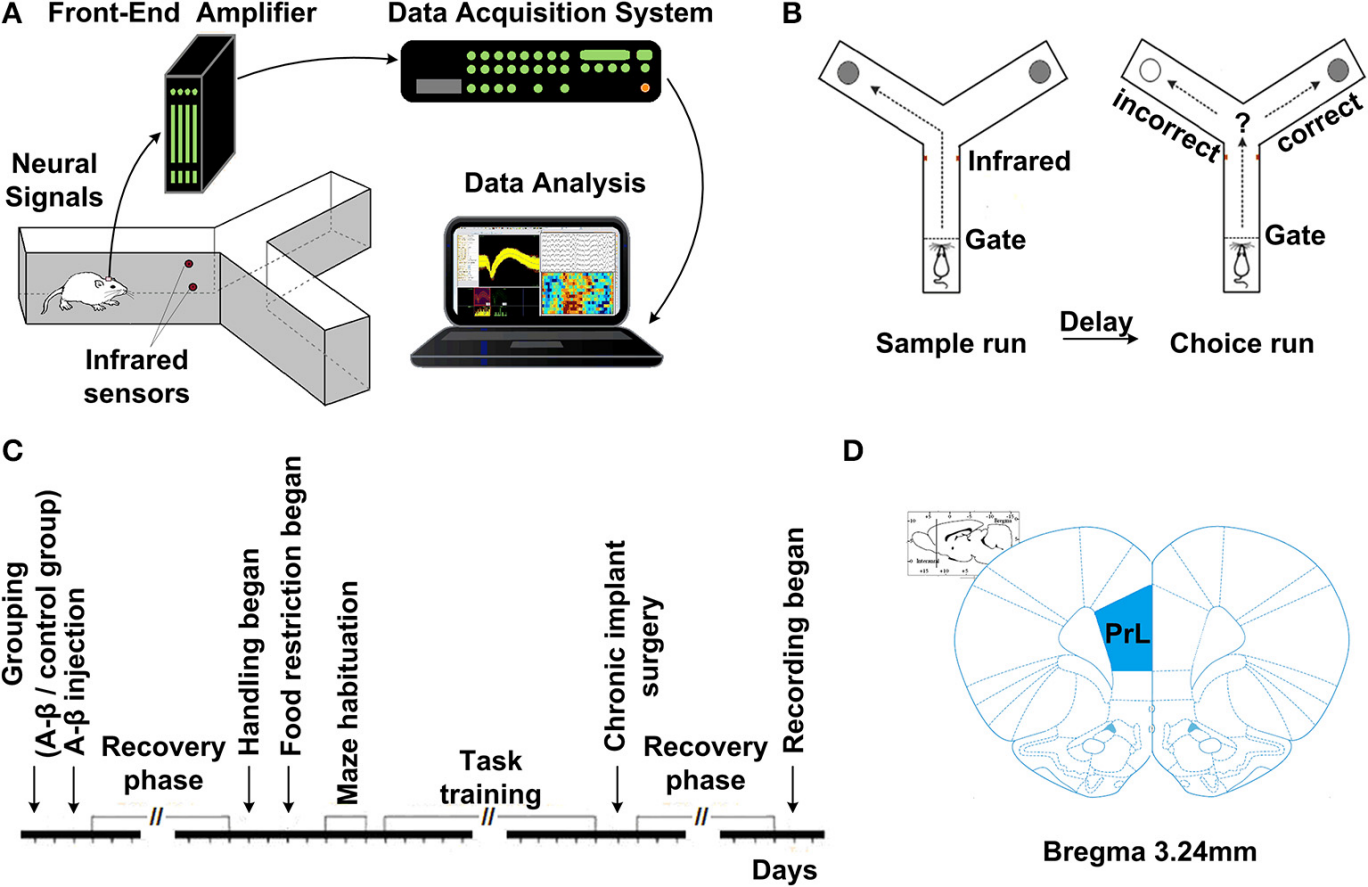
**Experiment setup and procedures. (A)** Diagram of experimental setup, consisting of a rat with implanted microelectrodes in a Y-maze, the Cerebus front-end amplifier, the Cerebus data acquisition system and analysis software. **(B)** Schematic representation of the delayed-alternation task in Y-maze. Dashed lines represent the locations of removable guillotine doors that restricted access to different parts of the maze. Food cups, which are used for reward delivery, are located at the ends of two arms. Arrows show possible correct paths. Each trial consisted of a sample run and a choice run. On a sample run, both the left- and right-arms of the maze are baited and rats could get food by entering into either of them. After a 5 s delay, the rats would have a choice run. Rats have to avoid the arm visited in the sample run and enter into the opposite arm to get reward. **(C)** Time line of the various phases of experiment. **(D)** Diagram of coronal sections showing the level and position of the medial prefrontal cortex, taken from the *Stereotaxic Coordinates* (Paxinos and Watson, 2005). The number indicates the antero-posterior coordinates caudal to bregma.

Fourteen days after Aβ_1−42_/salineinjection, all the rats were trained on a delayed-alternation Y-maze task. First, the rats were acclimatized to the handling procedures for 2 days. Next, food access was limited to 2 h per day. The quantity of food was adjusted to maintain body weights at 85% of individual baseline free-feeding weights, adjusted for growth. Water was freely available in cages. Following habituation, the groups of rats were trained on a delayed-alternation task in Y-maze. The rats were given two training sessions (10 trials per session) per day. Each trial consisted of a sample run and a choice run. On the sample run, the rats were allowed to go either left or right to get a small piece of food reward in the food well at the end of the arm. After consuming the reward, the rat voluntarily went back to the start of Y-maze. After 5 s delay, the rats would have a “choice run.” The rats were rewarded for choosing the previously unvisited arm. After completing a trial, the rats were allowed to return and start next trial. To perform the task correctly, rats had to remember which arm had been visited in the previous trial and select the opposite one. For the control group, electrophysiological recording was initiated once rats' performance was stable at ~80% correct on two consecutive days. For the Aβ group, regardless of whether the rats could reach the criterion, the rats received chronic implants after same training sessions as control. The experimental procedures are shown in Figure 1.

### Electrophysiological recordings and data processing

After training, all the rats underwent a chronic implant surgery. The coordinates for PFC were determined according to the rat brain atlas in stereotaxic coordinates (mPFC, anterior, 2.5–4.5 mm; lateral, 0.2–1.0 mm; horizontal, 2.5–3.0 mm from bregma). 16-channel microelectrode arrays (2^*^0.3 mm, with 0.25 mm interelectrode spacing, nickel-chromium, <1 MΩ) were implanted into rat mPFC under aseptic conditions and chloral hydrate (350 mg/kg) anesthesia.

**Figure 2:**
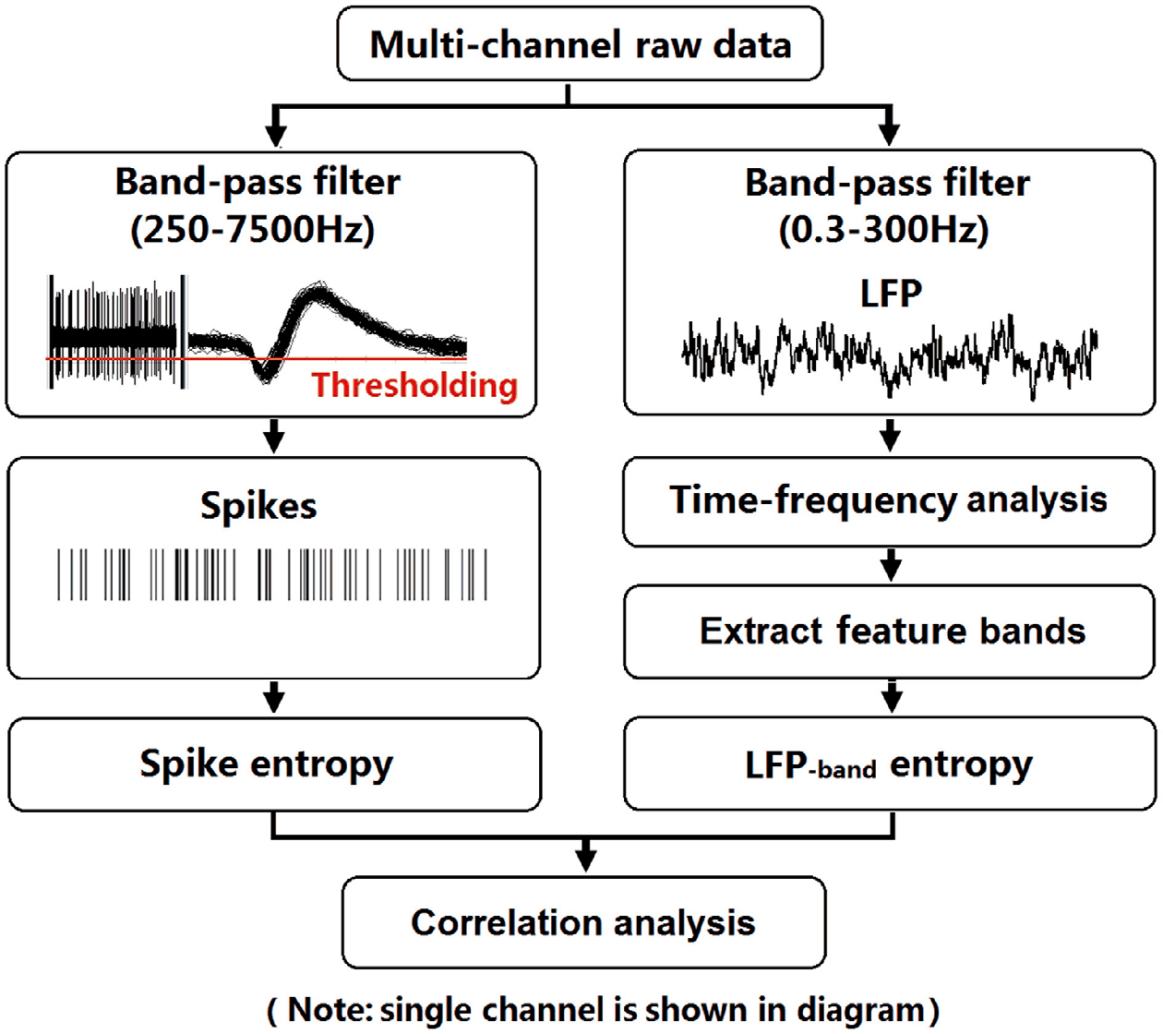
**Flow chart for data analysis**. To illustrate the procedure, we selected an arbitrary single channel to display. LFPs (band-pass filter: 0.3–300 Hz) were obtained from raw data via digital filters. Feature bands were further extracted according to the results of time-frequency spectral analysis. Spikes (band-pass filter: 250–7500 Hz) exceeding a preset voltage threshold were sampled at 30 kHz and were stored with time stamps. Then, entropy estimation of LFPs and spikes were performed. Finally, the correlation of spike entropy and LFP entropy was calculated to assess the spike-LFP coordination.

After recovery, neural activity was recorded while rats again performed the delayed-alternation Y-maze task. Wideband neural signals were recorded with a Cerebus Data Acquisition System (Cyberkinetics, USA). The timing of behavioral events was marked online by an infrared sensor in the Y-maze. Time 0 indicates the moment when the rat was at the choice point on Y-maze, which was measured by an infrared sensor. Local field potentials (low-pass filter: 0.3–300 Hz) were extracted via digital filters in the Neural Signal Processor. Spikes (high-pass filter: 250–7500 Hz) exceeding a preset voltage threshold were sampled at 30 kHz per channel and were stored with time stamps. Units with low signal-to-noise (<3.0) or a very low baseline firing rate (<30 spikes/min) were discarded. The data analysis workflow is shown in Figure 2.

### Data analysis and statistics

We recorded spikes and LFPs from 8 rats (4 control group; 4 Aβ group) for 2 sessions using 16-channel implanted electrode arrays while they performed the Y-maze working memory task. Time 0 indicates the moment when the rat was at the choice point on Y-maze, which was measured by an infrared sensor. In total, we describe 122 trials (66 trials with control and 56 trials with Aβ rats) in the present paper. Data in the text and figures are expressed as means ± s.e.m. Statistical differences were evaluated by using ANOVA and *t*-test (Student's *t*-test/Welch-Satterthwaite *t*-test). Specifically, behavioral accuracy was analyzed using ANOVA. Comparisons of entropy and spike-LFP coordination between the two groups were done by using *t*-test. Comparisons of entropy and spike-LFP coordination between correct and incorrect trials were evaluated by using *t*-test. *P*-values are marked statistically significant as follows: ^*^P < 0.05, ^**^P < 0.01.

#### Time-frequency spectral analysis

Recorded LFPs were first filtered by a 50 Hz notching filter and baseline drifts were removed. Spectral analysis was used to assess the dominant frequencies in the LFPs during the task. All spectra are calculated using multi-tapers. To illustrate the temporal modulation of power in different frequency bands, the LFP spectrum was estimated on a 500-ms window (125-ms moving step, 1 Hz resolution). Then the sub-bands were obtained by filtering the raw LFPs.

#### Entropy estimation of spikes and LFPs

The variability of LFP (in particular theta and gamma bands) and spikes were quantified by estimating Shannon entropy. The mathematical computation of entropy has been described in a previous paper (Shannon, 1948). When estimating the LFP entropy on each trial, we used a sliding window (500 ms) with a moving step (125 ms). And in each window, we got a distribution of LFP amplitudes in each channel. The entropy of LFP in each channel was estimated and then averaged over channels.

In each window, the amplitudes of LFPs were binned and the distribution of amplitude values was estimated with a histogram. The number of amplitudes in the *i*-th bin was counted (*A*_*i*_) and the probability of amplitude values in the *i*-th bin (*i* = 1, 2, …, *n*) calculated as following:

(1) $$p_{i}=A_{i}/\sum_{i\;=\;1}^{n}A_{i}$$

Entropy of the LFP amplitude values distribution was defined using Shannon's formula:

(2) $$H(X)=-\sum_{i\;=\;1}^{n}p_{i}\log p_{i}$$

where *H*(*X*) denotes the entropy of LFP amplitude distribution, and *P*_*i*_ denotes the relative frequency of the *i*-th bin. The entropy of LFP in each channel was estimated and then averaged over different channels. The same method was used to compute the entropy of theta- and gamma- band LFPs.

To estimate the entropy of spikes (representing the randomness of the distribution of population spiking), the inter-spike intervals (ISIs) were measured and the distribution of ISIs was estimated in a histogram with a 0.5 ms bin width. The entropy of the spike train in each channel was estimated and averaged over channels. Then the number of spikes in the *i*-th bin was counted (*S*_*i*_) and the firing probability of the *i*-th bin (*i* = 1, 2, …, *n*) was calculated:

(3) $$p_{i}=S_{i}/\sum_{i\;=\;1}^{n}S_{i}$$

The entropy of the spiking was computed using Shannon's formula:

(4) $$H(X)=-\sum_{i\;=\;1}^{n}p_{i}\log p_{i}$$

where *H*(*X*) denotes the entropy of spiking distribution, and *P*_*i*_ denotes the firing probability of the *i*-th bin. The entropy values of the spikes and LFPs were estimated in a selected window (500 ms) with a moving step (125 ms) across the entire task.

#### Estimation of spike-LFP correlation based on entropy

The coordination between spikes and LFPs were assessed via the Pearson correlation between spike entropy and LFP entropy. This was done separately for each trial, and for the theta and gamma bands.

We estimated the correlation between the spike entropy and LFP entropy across the entire task using the formula as follows:

(5) $$r_{XY}=\frac{\sum_{i\;=\;1}^{N}\left(X_{i}-\bar{X}\right)(Y_{i}-\bar{Y})}{\sqrt{\sum_{i\;=\;1}^{N}{\left(X_{i}-\bar{X}\right)}^{2}}\sqrt{\sum_{i\;=\;1}^{N}{\left(Y_{i}-\bar{Y}\right)}^{2}}}$$

where *i* denotes the *i*-th window and *X_i_*, *Y_i_* denotes the spike entropy and LFP entropy in the *i*-th window. The entropy correlation of spikes and LFPs were calculated in 500-ms windows across the entire task.

## Results

### Immunohistochemistry on Aβ_1−42_-induced toxicity model

**Figure 3:**
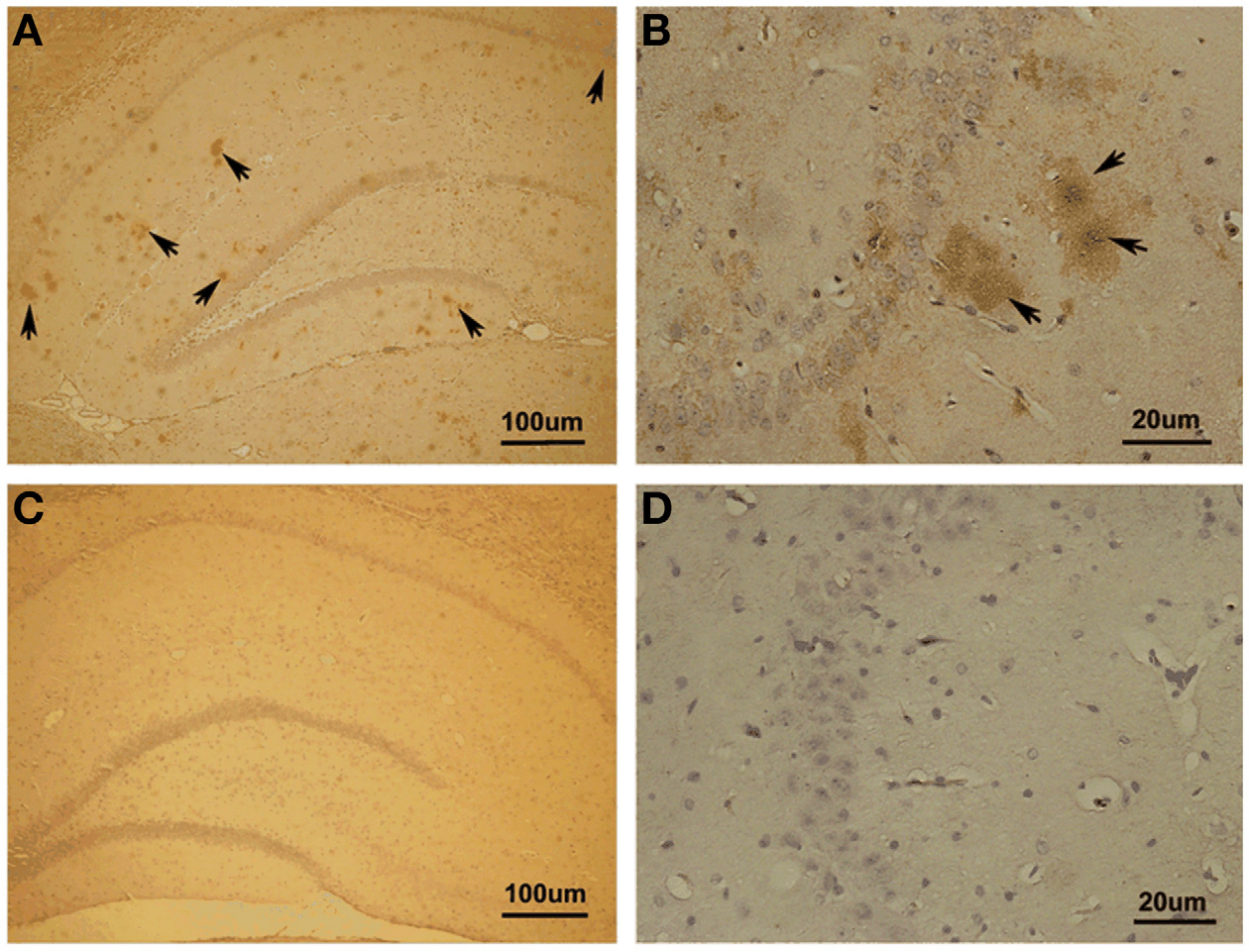
**Immunohistochemistry in Aβ_1–42_-induced toxicity model and control. (A,B)** Aβ_1–42_-induced toxicity model. **(C,D)** Control group. Arrows showed Aβ_1–42_-positive immunostaining area of hippocampus. Amyloid plaques depositions in hippocampus of Aβ injection group were observed.

First, we investigated whether Aβ_1−42_ could be detected in the hippocampus with Aβ_1−42_ intra-hippocampal injection. Using immunohistochemistry, we found a number of Aβ_1−42_ deposits in the hippocampus (Figure 3). The results indicated that the incubated Aβ_1−42_ had been successfully injected into rat hippocampus and amyloid plaques depositions were observed.

Notably, we noticed the Aβ diffusion after Aβ injection in hippocampus. To clarify the extent of Aβ diffusion, we further quantified Aβ deposition. The quantification was performed using the image processing system to analyze the area occupied by positive immunostaining. We found the most (85.07 ± 1.11% of total positive areas) Aβ deposits in the dorsal and ventral hippocampus as well as a small amount (14.93 ± 1.11%) deposits outside of the hippocampus proper, in the lateral habenula (LHb) and thalamus.

### Behavioral performance

**Figure 4:**
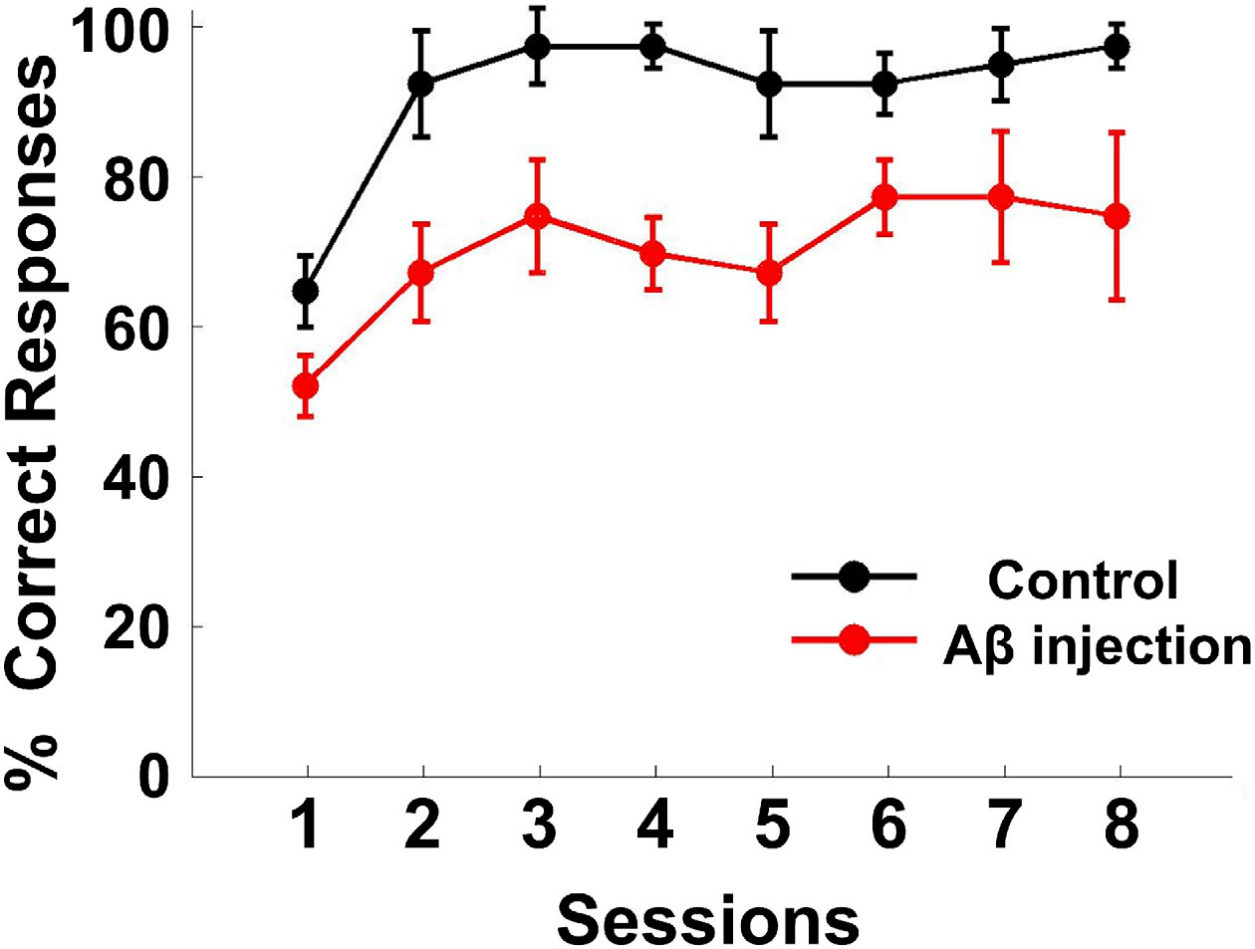
**Behavioral performance**. Task performance rates of the delayed alternate choice for the two groups. The curves show the mean percentage of correct responses in the Aβ group (red) and control group (black). The error bars indicate s.e.m.

The performance of each rat in each training session was measured by the accuracy of responses (percent correct, Figure 4). The response accuracy in the Aβ group was significantly worse than the control group [ANOVA, *F*_(1, 6)_ = 82.620, *P* < 0.05]. The percentage of correctly completed trials increased as training progressed in both control (black) and Aβ group (red). For the control group, in eight training sessions, the behavioral performance gradually reached a criterion level of performance (80% correct for two consecutive days). However, none of the rats from the Aβ group reached the criterion. During the recording sessions, the response accuracy in the Aβ group (64.47 ± 3.82%) was also worse than the control group (86.11± 5.77%) (*t*-test, *t* = 3.608, *P* < 0.05).

### Power changes of LFPs in rat mPFC during the working memory task

**Figure 5:**
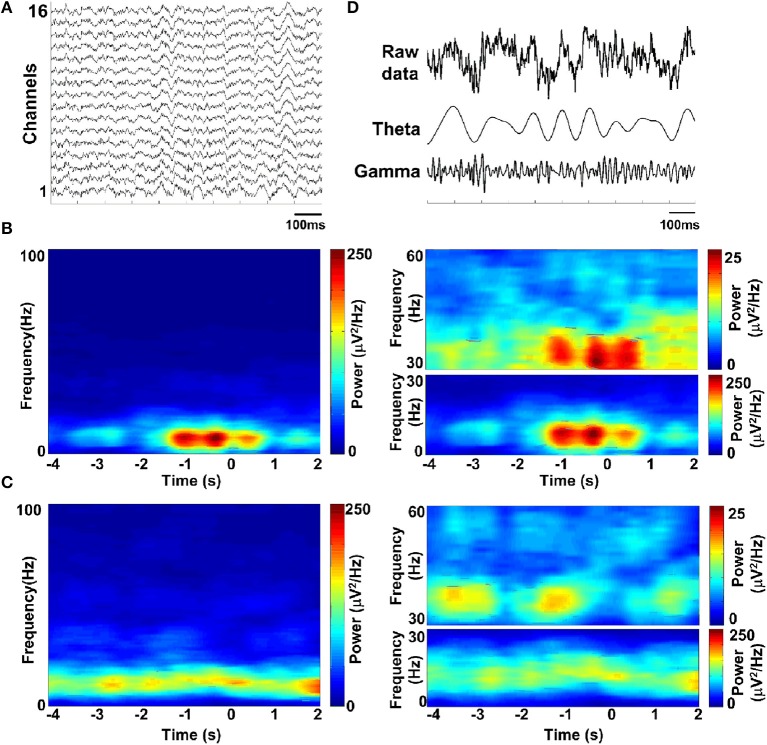
**LFP spectrograms during the working memory task. (A)** Raw data recorded from rat PFC by using multi-electrodes. **(B)** Time-frequency power spectrum in a trial when rat got success. Shown (**B**, left) is the averaged power spectrum across channels (*n* = 16). Time is on the x-axis; frequency is on the y-axis. Power is indicated by color. Time 0 indicates the moment when the rat was at the choice point on Y-maze, which was measured by an infrared sensor. Since the absolute power of theta and gamma band differed greatly, we assigned separate scales for illustration (**B**, right). **(C)** Time-frequency power spectrum in a failed trial. **(D)** Band-pass filtered LFPs, filtered in the theta-band (4–12 Hz) and gamma-band (30–60 Hz) (from top to bottom; raw data, theta and gamma).

Spectral analysis was used to assess the dominant frequencies in the LFPs during the task. To illustrate the temporal modulation of power in different frequency bands, the LFP spectrum was estimated on a 500-ms window with 1 Hz resolution. Figure 5A shows exemplar LFP of a single recording site during the working memory task. Time-frequency power spectrums are illustrated for two trials when the rat succeeded (Figure 5B) and when the rat failed (Figure 5C). As can be seen in Figure 5B, theta power was much larger than gamma power all through the task. Interestingly, both theta and gamma power increased during correct trials. However, on incorrect trials, the power was much lower than on correct trials and the theta and gamma power did not increase during the trials. The sub-bands were obtained by band-pass filtering (theta: 4–12 Hz; gamma: 30–60 Hz, shown in Figure 5D).

### Dynamic entropy of neural activity in rat PFC during the working memory task

**Figure 6:**
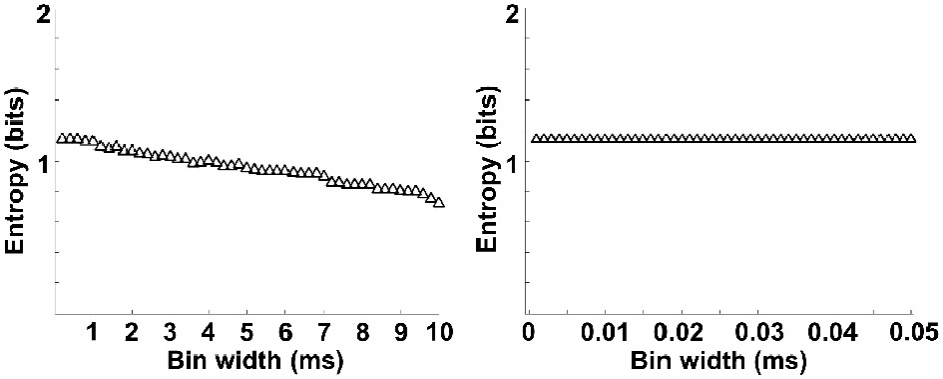
**Entropy estimation of spikes as a function of bin widths**. Entropy varies with bin size (left). The data is linearly extrapolated to the point that bin size is zero (right). Entropy values decreased slightly with the increasing bin size and entropy values did not change when the bin sizes <0.5 ms.

The entropy value strongly depends on the bin size, especially for the estimation of spike entropy. For the purpose of entropy estimation, a bin width of 0.5 ms was employed in the present paper, determined in accordance with the literature (Reeke and Coop, 2004). To assess the influence of bin width on the entropy value, we plotted the entropy value with different bin sizes and then linearly extrapolate the curve to the point that the bin size is zero, following the methods of Brenner et al. (2000). As can be seen from Figure 6, entropy values decreased slightly with the increasing bin size and entropy values did not change when the bin sizes <0.5 ms. Therefore, a bin width of 0.5 ms was employed.

**Figure 7:**
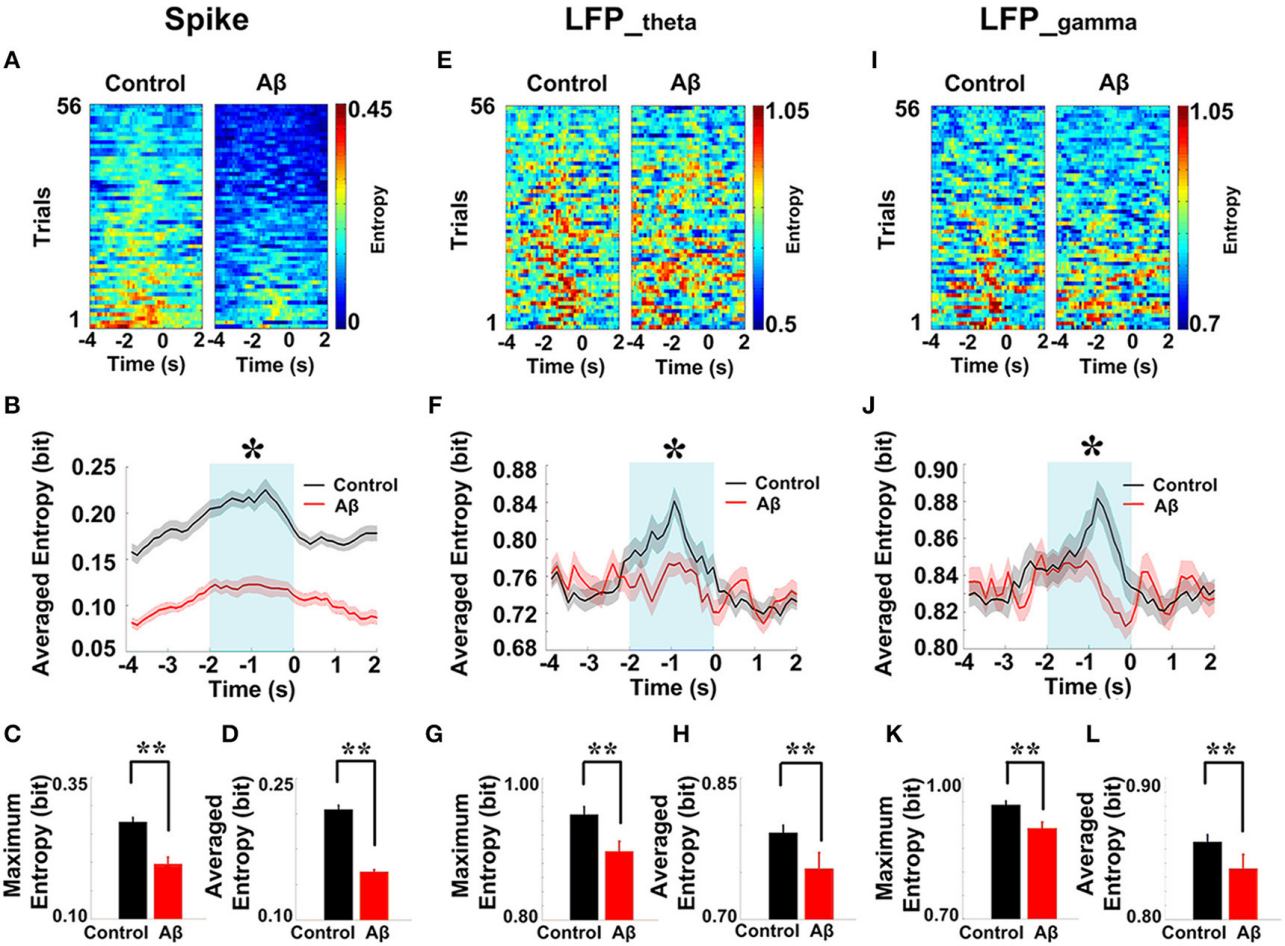
**Dynamic entropy of neural activity in rat PFC during the working memory task in the Aβ (*n* = 56 trials) and control (*n* = 56 trials) groups. (A)** Dynamic entropy of spikes from example trials (left: control; right: Aβ). **(B)** Averaged entropy of spikes across all trials in Aβ (red) and control (black) groups. The shaded region indicates s.e.m. The light blue area indicates the period used for statistical analysis. Averaged entropy values were compared between two groups in this time window (^*^P < 0.05). **(C)** Peaks of entropy values (^*^P < 0.05, ^**^P < 0.01). **(D)** Average of entropy values. **(E)** Dynamic entropy of theta-band LFP. **(F)** Averaged entropy of theta band LFP across all trials in Aβ and control. **(G)** Peaks of entropy values. **(H)** Average entropy values. **(I)** Dynamic entropy of gamma band LFP. **(J)** Averaged entropy of gamma-band LFP across all trials. **(K)** Peaks of entropy values. **(L)** Average entropy values.

We then analyzed the dynamic entropy changes of neural activity in rat PFC during the working memory task in Aβ and control groups. In the control group, across the whole task time, the entropy values of spikes and LFPs (theta and gamma bands) were observed to increase up until the choice point, and then decline. The peaks of the entropy appeared before the choice point in the trials. However, there was no statistically significant change in the Aβ group (Figure 7). Further analyses were performed to compare the peaks and averages of entropy between the Aβ and control groups (Table 1). The peaks and averages of spike entropy in Aβ group were significantly lower than those in control (peak: *t*-test, *t* = 9.155, *P* < 0.01; average: *t*-test, *t* = 11.664, *P* < 0.01; Figures 7A–D). The peaks and averages of LFP_theta_ entropy in Aβ group were significantly higher than those in control (peak: *t*-test, *t* = 3.200, *P* < 0.01; average: *t*-test, *t* = 2.655, *P* < 0.01; Figures 7E–H). Moreover, the peaks and averages of LFP_gamma_ entropy in control were significantly higher than those in Aβ group (peak: *t*-test, *t* = 4.224, *P* < 0.01; average: *t*-test, *t* = 4.437, *P* < 0.01; Figures 7I–L).

**Table 1:** **Entropy of neural activity during working memory task in the Aβ and control groups**.

	**Aβ**		**Control**	
	**Peak**	**Averaged**	**Peak**	**Averaged**
Spike entropy	0.197 ± 0.012^**^	0.118 ± 0.003^**^	0.271 ± 0.008	0.206 ± 0.006
LFP_theta_ entropy	0.897 ± 0.014^**^	0.754 ± 0.017^**^	0.949 ± 0.011	0.792 ± 0.008
LFP_gamma_ entropy	0.893 ± 0.013^**^	0.836 ± 0.010^**^	0.943 ± 0.008	0.855 ± 0.005

All entropy values in bit.

Data are shown as mean ± s.e.m.

** P < 0.01 for tests of significant difference.

### Estimation of spike-LFP coordination based on entropy

**Figure 8:**
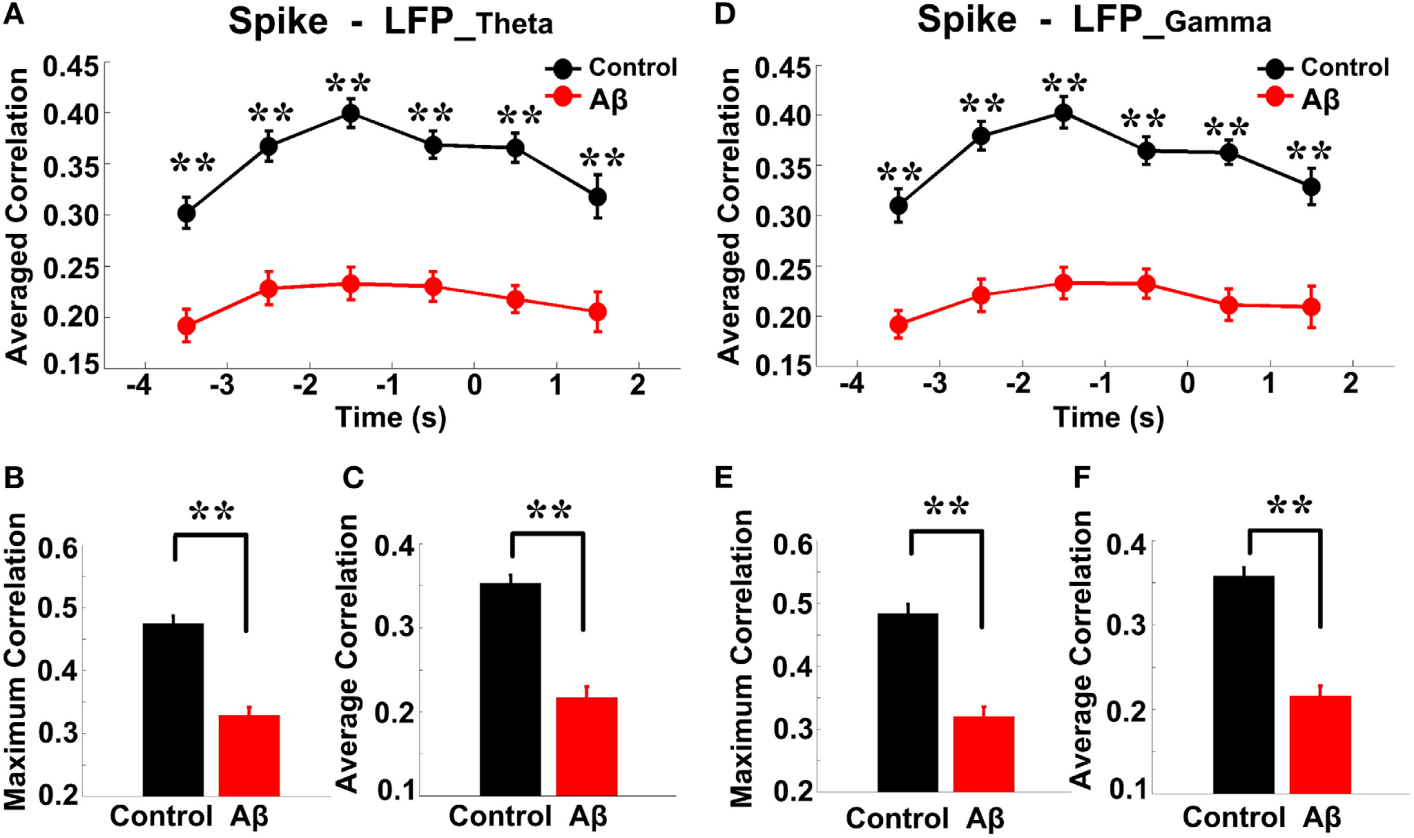
**Entropy correlation between spikes and LFPs during the working memory task in the Aβ (*n* = 56) and control (*n* = 56) groups. (A)** Averaged entropy correlations between spikes and theta band LFPs (black: control; red: Aβ). Data were averaged across all the trials within each group; error bars reflect one standard error of the mean. **(B)** Peaks of spike-LFP_theta_ correlation (^**^P < 0.01). **(C)** Average spike- LFP_theta_ correlation. **(D)** Averaged entropy correlations between spikes and gamma band LFPs. **(E)** Peaks of spike-LFP_gamma_ correlation. **(F)** Averages of spike-LFP_gamma_ correlation.

Spike-LFP coordination was assessed by estimating the correlation between spike entropy and LFP entropy, across the entire task time. Results in all panels (Figure 8) report data collected during the trials and show the average over the entire dataset. In the control group, across the whole task time, correlations (in both theta and gamma band) were observed to increase up until the choice point, and then to decrease. Moreover, the correlations were highest during the period from 2 to 0 s prior to the choice-point crossing. The spike-LFP_theta_ correlation in Aβ group was significantly lower than that in control group (peak: *t*-test, *t* = 8.648, *P* < 0.01; average: *t*-test, *t* = 9.954, *P* < 0.01; Figures 8A–C). Moreover, the spike-LFP_gamma_ correlation in control was significantly higher than that in Aβ group (peak: *t*-test, *t* = 9.823, *P* < 0.01; average: *t*-test, *t* = 9.731, *P* < 0.01; Figures 8D–F, Table 2).

**Table 2:** **Spike-LFP correlation during working memory task in the Aβ and control groups**.

	**Aβ**		**Control**	
	**Peak**	**Averaged**	**Peak**	**Averaged**
Spike-LFP_theta_	0.329 ± 0.015^**^	0.353 ± 0.010^**^	0.475 ± 0.012	0.217 ± 0.013
Spike-LFP_gamma_	0.320 ± 0.016^**^	0.216 ± 0.012^**^	0.484 ± 0.013	0.358 ± 0.010

Data are shown as mean ± s.e.m.

** P < 0.01 for tests of significant difference.

### Spike-LFP coordination on correct and incorrect trials in the control group

The overall success rates of the rats in the control group were generally high. We observed a stereotypical spike-LFP coordination: a pattern of increase, peak, and decline in correctly performed trials. To obtain insights into the nature of information conveyed in correct trials, we further analyzed the neuronal activity and compared the spike-LFP coordination between correct and incorrect trials.

**Figure 9:**
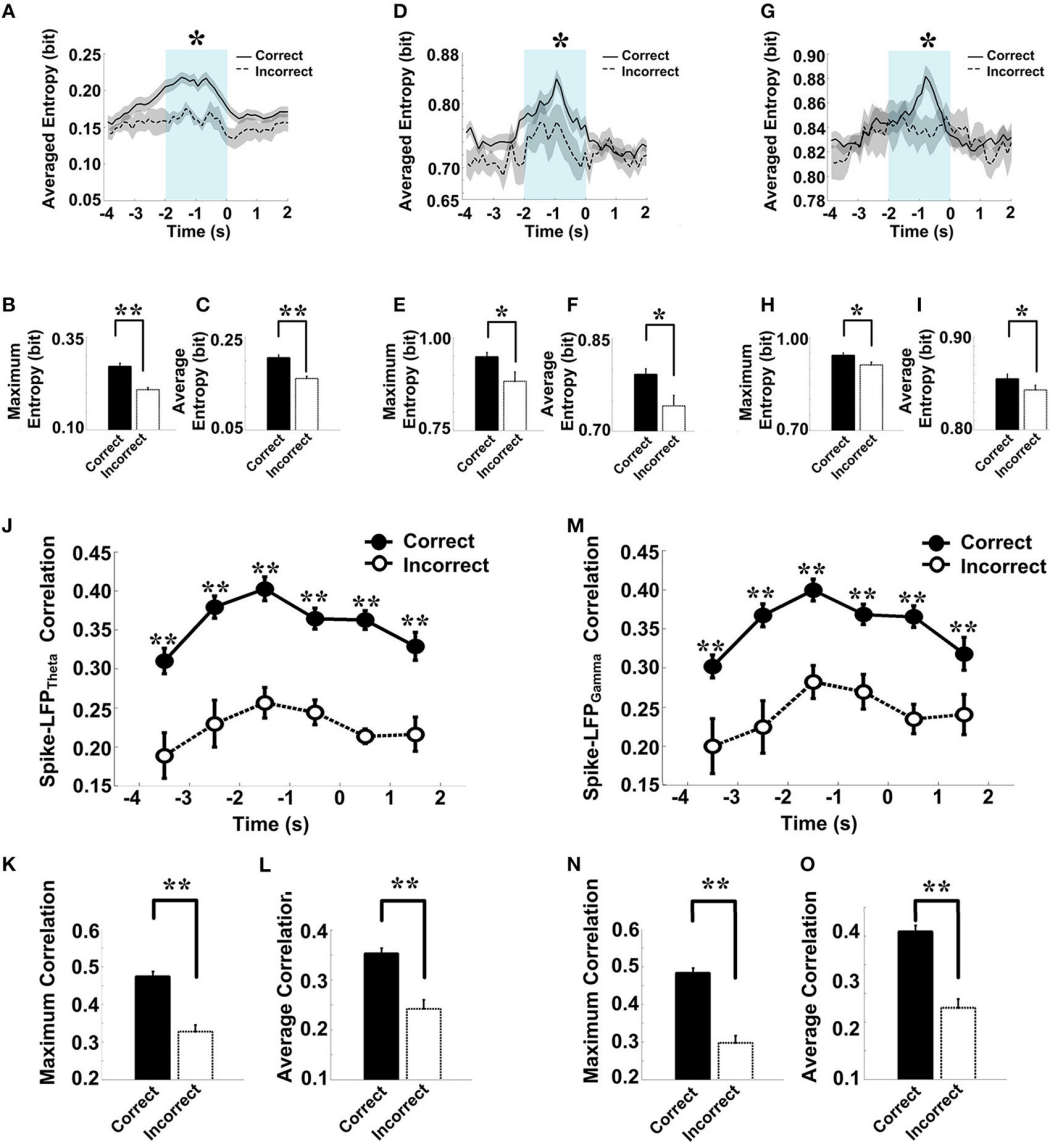
**Spike-LFP coordination in correct (*n* = 56) and incorrect (*n* = 10) trials in the control group. (A)** Averaged spikes entropy across correct (solid) and incorrect (dashed) trials. The shaded region indicates s.e.m. The light blue area indicates the period used for statistical analysis. Averaged entropy values were compared in this time window (^*^P < 0.05, ^**^P < 0.01). **(B)** Peaks of spike entropy. **(C)** Averages of spike entropy. **(D)** Averaged entropy of LFP_theta_ in correct and incorrect trials. **(E)** Peaks of LFP_theta_ entropy. **(F)** Averages of LFP_theta_ entropy. **(G)** Averaged entropy of LFP_gamma_ in correct and incorrect trials. **(H)** Peaks of LFP_gamma_ entropy. **(I)** Averages of LFP_gamma_ entropy. **(J)** Averaged spike-LFP_theta_ correlations in correct and incorrect trials (solid circle: correct; hollow circle: incorrect). Error bars reflect one standard error of the mean. **(K)** Peaks of spike-LFP_theta_ correlation. **(L)** Averages of spike-LFP_theta_ correlation. **(M)** Averaged spike-LFP_gamma_ correlations in correct and incorrect trials. **(N)** Peaks of spike-LFP_gamma_ correlation. **(O)** Averages of spike- LFP_gamma_ correlation.

As shown in Figure 9, in correct trials (*n* = 56), the entropy of spikes/LFPs were observed to increase up until the choice point, and then decline. The peaks appeared before the choice point. However, there was no significant difference in the entropy of spikes/LFPs in the incorrect trials (*n* = 10). Further statistic analyses were performed to compare the peaks and averages of entropy between the correct and incorrect trials (Table 3). The spike entropy values in correct trials were significantly higher than those in incorrect trials (peak: *t*-test, *t* = 3.124, *P* < 0.01; average: *t*-test, *t* = 2.903, *P* < 0.01; Figures 9A–C). The LFP_theta_ entropy and LFP_gamma_ entropy in correct trials were also higher than those in incorrect trials (theta: peak: *t*-test, *t* = 2.287, *P* < 0.05; average: *t*-test, *t* = 2.242, *P* < 0.05; Figures 9D–F; gamma: peak: *t*-test, *t* = 2.142, *P* < 0.05; average: *t*-test, *t* = 2.128, *P* < 0.05; Figures 9G–I).

**Table 3:** **Entropy of neural activity in correct and incorrect trials in the control group**.

	**Correct**		**Incorrect**	
	**Peak**	**Averaged**	**Peak**	**Averaged**
Spike entropy	0.271 ± 0.008	0.206 ± 0.006	0.208 ± 0.006^**^	0.161 ± 0.005^**^
LFP_theta_ entropy	0.949 ± 0.011	0.792 ± 0.008	0.883 ± 0.025^*^	0.741 ± 0.017^*^
LFP_gamma_ entropy	0.943 ± 0.008	0.855 ± 0.005	0.912 ± 0.009^*^	0.843 ± 0.005^*^

All entropy values in bit.

Data are shown as mean ± s.e.m.

* *P < 0.05*,

** P < 0.01 for tests of significant difference.

In the control group, the spike-LFP coordination in correct trials was observed to increase up until the choice point, and then to decline. However, no significant change was found in the correlation for the incorrect trials (Table 4). The spike-LFP_theta_ correlations in correct trials were significantly higher than those in incorrect trials (peak: *t*-test, *t* = 5.183, *P* < 0.01; average: *t*-test, *t* = 4.489, *P* < 0.01; Figures 9J–L). The spike-LFP_gamma_ correlations in control were also higher than those in incorrect trials (peak: *t*-test, *t* = 5.923, *P* < 0.01; average: *t*-test, *t* = 5.408, *P* < 0.01; Figures 9M–O). Since increasing tendencies of spike-LFP coordination were found in correct trials while there was no significant change in incorrect trials, we propose that the strengthened spike-LFP coordination could be necessary for information manipulation in working memory.

**Table 4:** **Spike-LFP correlation in correct and incorrect trials in the control group**.

	**Correct**		**Incorrect**	
	**Peak**	**Averaged**	**Peak**	**Averaged**
Spike-LFP_theta_	0.475 ± 0.012	0.353 ± 0.010	0.328 ± 0.017^**^	0.242 ± 0.018^**^
Spike-LFP_gamma_	0.484 ± 0.013	0.358 ± 0.010	0.298 ± 0.019^**^	0.225 ± 0.015^**^

Data are shown as mean ± s.e.m.

** P < 0.01 for tests of significant difference.

### Spike-LFP coordination on correct and incorrect trials in the Aβ group

**Figure 10:**
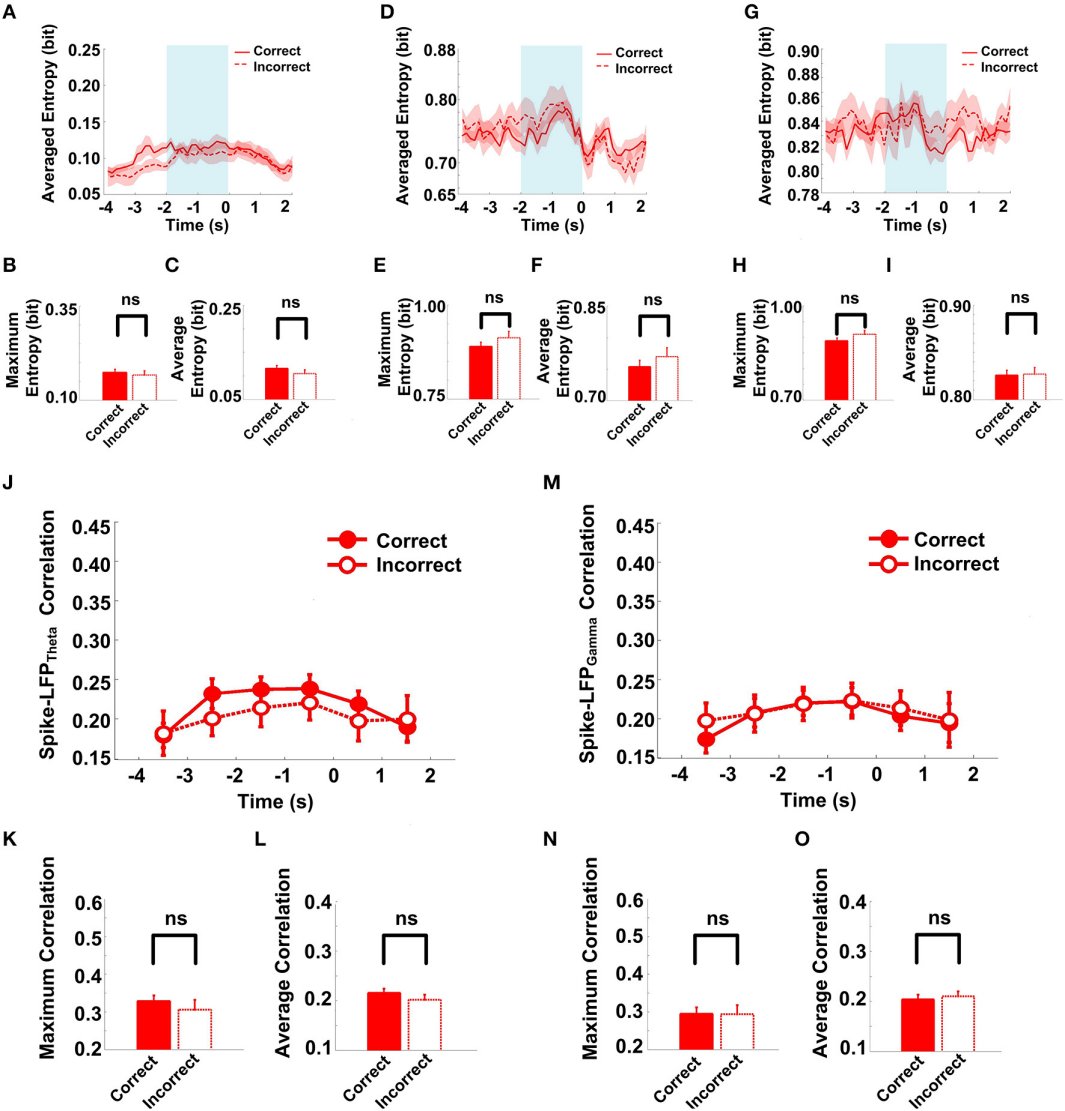
**Spike-LFP coordination in correct (*n* = 35) and incorrect (*n* = 21) trials in the Aβ group. (A)** Averaged spikes entropy across correct (solid) and incorrect (dashed) trials. The shaded region indicates s.e.m. The light blue area indicates the period used for statistical analysis. Averaged entropy values were compared in this time window. **(B)** Peaks of spike entropy. **(C)** Averages of spike entropy. **(D)** Averaged entropy of LFP_theta_ in correct and incorrect trials. **(E)** Peaks of LFP_theta_ entropy. **(F)** Averages of LFP_theta_ entropy. **(G)** Averaged entropy of LFP_gamma_ in correct and incorrect trials. **(H)** Peaks of LFP_gamma_ entropy. **(I)** Averages of LFP_gamma_ entropy. **(J)** Averaged spike-LFP_theta_ correlations in correct and incorrect trials (solid circle: correct; hollow circle: incorrect). Error bars reflect one standard error of the mean. **(K)** Peaks of spike-LFP_theta_ correlation. **(L)** Averages of spike-LFP_theta_ correlation. **(M)** Averaged spike-LFP_gamma_ correlations in correct and incorrect trials. **(N)** Peaks of spike- LFP_gamma_ correlation. **(O)** Averages of spike-LFP_gamma_ correlation.

The success rates of the rats in the Aβ group were relatively low and as the spike-LFP coordination showed no statistically significant change. To determine whether the spike-LFP coordination is associated with correct/incorrect performances, we further compared the spike-LFP coordination on correct and incorrect trials. In the Aβ group, as shown in Figure 10, there was no statistically significant difference in spikes/LFPs entropy between the correct and incorrect trials.

The spike entropy in correct (*n* = 35) and incorrect (*n* = 21) trials showed no significant difference (peak: *t*-test, *t* = 0.453, *P* > 0.05; average: *t*-test, *t* = 1.039, *P* > 0.05). The LFP_theta_ entropy (peak: *t*-test, *t* = 1.271, *P* > 0.05; average: *t*-test, *t* = 0.846, *P* > 0.05) and LFP_gamma_ entropy (peak: *t*-test, *t* = 1.588, *P* > 0.05; average: *t*-test, *t* = 0.366, *P* > 0.05) in correct and incorrect trials showed no significant difference (Table 5). Moreover, the spike-LFP correlations in correct and incorrect trials showed no significant difference (theta: peak: *t*-test, *t* = 0.453, *P* > 0.05; average: *t*-test, *t* = 1.039, *P* > 0.05; gamma: *t*-test, *t* = 0.050, *P* > 0.05; average: *t*-test, *t* = 0.250, *P* > 0.05; Table 6). The results indicated that the spike-LFP coordination showed no significant difference, whether the rats in Aβ group performed the task correctly or incorrectly.

**Table 5:** **Entropy of neural activity in correct and incorrect trials in the Aβ group**.

	**Correct**		**Incorrect**	
	**Peak**	**Averaged**	**Peak**	**Averaged**
Spike entropy	0.174 ± 0.008	0.116 ± 0.006	0.167 ± 0.011	0.105 ± 0.008
LFP_theta_ entropy	0.890 ± 0.012	0.754 ± 0.010	0.913 ± 0.017	0.770 ± 0.014
LFP_gamma_ entropy	0.889 ± 0.009	0.826 ± 0.005	0.910 ± 0.012	0.827 ± 0.007

All entropy values in bit.

Data are shown as mean ± s.e.m.

**Table 6:** **Spike-LFP correlation in correct and incorrect trials in the Aβ group**.

	**Correct**		**Incorrect**	
	**Peak**	**Averaged**	**Peak**	**Averaged**
Spike-LFP_theta_	0.329 ± 0.015	0.216 ± 0.007	0.306 ± 0.026	0.202 ± 0.010
Spike-LFP_gamma_	0.295 ± 0.018	0.204 ± 0.008	0.294 ± 0.024	0.210 ± 0.010

Data are shown as mean ± s.e.m.

## Discussion

Our results show that the spike-LFP coordination in the control group strengthened during working memory and the spike-LFP coordination in the Aβ group was weaker than that in the control group. We propose that the strengthened spike-LFP coordination is necessary for information manipulation in working memory and the Aβ-induced incoordination between spikes and LFPs causes the working memory impairment. Multi-disciplinary research has implicated Aβ with the cognitive impairments in AD and the mechanistic understanding of the ability of Aβ to interfere with synaptic plasticity and memory yields important insights into the pathophysiology of AD. Therefore, the findings on incoordination between spikes and LFPs may open a new perspective for mechanistic investigation of working memory deficits in AD.

Notably, Aβ was injected into dorsal hippocampus (dHPC) and were observed in dorsal and ventral hippocampus (dHPC and vHPC) and in LHb and thalamus. Since Aβ deposit was not shown in PFC, how this could affect spike-LFP coordinations in PFC?

As the most Aβ deposits were found in hippocampus after Aβ injection, the neuronal activity in mPFC was primarily affected by the alteration of hippocampus function. Accumulating evidence has suggested the hippocampal-PFC interaction is critical for successful task performance (Jones and Wilson, 2005; Izaki et al., 2008; Hyman et al., 2010; Sigurdsson et al., 2010; O'Neill et al., 2013). It is well recognized that the vHPC directly projects to the PFC (Hoover and Vertes, 2007). Inactivating the vHPC led to a reduction in hippocampal- PFC coherence and impaired working memory function (O'Neill et al., 2013). However, the dHPC does not project directly to the PFC (Gordon, 2011). The information from the dHPC must arrive at the mPFC through an indirect route and the vHPC may facilitate spatial working memory by relaying spatial information from the dHPC to the mPFC. A recent study has identified that the dHPC can affect the mPFC through indirect pathways (Zingg et al., 2014), which can explain how Aβ injection in the dHPC alters the neuronal functioning in the PFC.

Meanwhile, we also noticed a small amount deposits outside of the hippocampus proper, in the LHb and thalamus. These two structures are known to play roles in memory and are connected to the PFC, so the Aβ deposits in the two structures may induce the mPFC alteration. Since the information processing in cortico-hippocampal areas in cognitive behaviors is influenced by various neuromodulators (including dopamine, serotonin etc.) and the release of these neuromodulators is under the control of the LHb (Lecourtier and Kelly, 2007; Hikosaka, 2010), the dysfunction in LHb may induce the cognitive impairments (Lecourtier et al., 2004, 2006). On the other hand, thalamus links the multiple pathways in multiple cognitive processes and some thalamic neurons project to the PFC, so the thalamus is also in a good position to influence the PFC activity (Otake and Nakamura, 1998; Sesack and Grace, 2010). Therefore, the dysfunction in LHb and thalamus might partly have contributed to the PFC alteration.

Another interesting result in this study is the lack of differential modulation between correct and incorrect choices in Aβ injected animals, compared to the healthy rats. For the control group, the spike-LFP coordination strengthened on the correct trials. The results were not completely surprising since working memory has long been linked with an increase of power in the theta- and gamma-band in human hippocampus and cortex (Tesche and Karhu, 2000; Bastiaansen et al., 2002; Düzel et al., 2010) and monkey extrastriate visual cortex (Lee et al., 2005). Successful memory encoding is associated with increased synchronization of theta- and gamma-band oscillations in human (Friese et al., 2012). In particular, the activation of working memory is characterized by both increases in theta- and gamma-band synchronization in EEGs (Klimesch, 1996) and MEGs in human (Stam et al., 2002). Moreover, a previous study has reported strengthened spike-LFP coordination in healthy rats during working memory (Li et al., 2014). The results in the present paper are consistent with these previous findings. The results also revealed weaker spike-LFP coordination on incorrect trials, compared with correctly performed trials. Moreover, the spike-LFP coordination experienced the pattern of increase, peak, and decline on correct trials. By contrast, there was no significant difference on incorrect trials. However, for the Aβ group, the spike-LFP coordination showed no significant difference on either correct or incorrect trials. A possible explanation is that, for healthy subjects, the stronger spike-LFP coordination successfully encoded working memory information on correct trials while the weaker spike-LFP coordination failed to encode on the incorrect trials. However, for the Aβ-injected rats, the Aβ deposits induced synaptic plasticity failure (Small et al., 2001; Ma and Klann, 2012) in hippocampus and caused the dysfunction of hippocampal- PFC circuit, which further contributed to the lack of modulation in spike-LFP coordination.

In summary, our results demonstrate that the spike-LFP coordination in healthy subjects increases during the working memory task. In contrast, the spike-LFP coordination in Aβ group was weaker and did not significantly change across the entire task. The incoordination between spikes and LFPs in working memory deficits may thus provide a potential mechanism for cognitive deficits in AD.

## Author contributions

Xin Tian designed the experiment; Wenwen Bai, Tiaotiao Liu, Hu Yi, and Jing Wei carried out the experiments; Wenwen Bai analyzed the data; Wenwen Bai and Xin Tian wrote the manuscript. All authors read and approved the final manuscript.

### Conflict of interest statement

The authors declare that the research was conducted in the absence of any commercial or financial relationships that could be construed as a potential conflict of interest.
